# Contribution of the subthalamic nucleus to motor, cognitive and limbic processes: an electrophysiological and stimulation study in monkeys

**DOI:** 10.3389/fnins.2024.1257579

**Published:** 2024-02-22

**Authors:** Mathilde Bertrand, Stephan Chabardes, Vincent Fontanier, Emmanuel Procyk, Julien Bastin, Brigitte Piallat

**Affiliations:** ^1^Univ. Grenoble Alpes, Inserm, U1216, Grenoble Institute of Neurosciences, Grenoble, France; ^2^Univ. Grenoble Alpes, Department of Neurosurgery, Inserm, U1216, CHU Grenoble Alpes, Grenoble Institute Neurosciences, Grenoble, France; ^3^Clinatec-CEA Leti, Grenoble, France; ^4^Univ. Lyon 1, Inserm, Stem Cell and Brain Research Institute U1208, Bron, France; ^5^Medinetic Learning, Research Department, Paris, France

**Keywords:** subthalamic nucleus, deep brain stimulation, behavioral task, non-human primate, electrophysiology

## Abstract

Deep brain stimulation of the subthalamic nucleus (STN) has become the gold standard surgical treatment for Parkinson’s disease and is being investigated for obsessive compulsive disorders. Even if the role of the STN in the behavior is well documented, its organization and especially its division into several functional territories is still debated. A better characterization of these territories and a better knowledge of the impact of stimulation would address this issue. We aimed to find specific electrophysiological markers of motor, cognitive and limbic functions within the STN and to specifically modulate these components. Two healthy non-human primates (*Macaca fascicularis*) performed a behavioral task allowing the assessment of motor, cognitive and limbic reward-related behavioral components. During the task, four contacts in the STN allowed recordings and stimulations, using low frequency stimulation (LFS) and high frequency stimulation (HFS). Specific electrophysiological functional markers were found in the STN with beta band activity for the motor component of behavior, theta band activity for the cognitive component, and, gamma and theta activity bands for the limbic component. For both monkeys, dorsolateral HFS and LFS of the STN significantly modulated motor performances, whereas only ventromedial HFS modulated cognitive performances. Our results validated the functional overlap of dorsal motor and ventral cognitive subthalamic territories, and, provide information that tends toward a diffuse limbic territory sensitive to the reward within the STN.

## Introduction

Interactions between animals and their environment is achieved through behavior and controlled by cerebral structures. Among them, the basal ganglia contribute to the selection of motor, cognitive and limbic components that define behaviors. As part of the basal ganglia network, the subthalamic nucleus (STN), with its direct connections to the cortex, plays a crucial role in goal-directed behavior (Albin et al., 1989). The STN involvement in the motor component has been largely documented from rodents to non-human primates (NHPs). STN focal inhibition with GABA receptor agonists induced postural asymmetry in rodents (Dybdal and Gale, 2000), and dyskinetic movements or motor stereotypies when injected in the middle part of the STN in NHPs (Hamada and DeLong, 1992; Karachi et al., 2009). More recently, a study in mice using selective optogenetic techniques has demonstrated that inhibition of the STN enhances locomotion while its excitation reduces it (Guillaumin et al., 2021). Other preclinical studies have highlighted the STN cognitive and limbic roles. Naïve rats with STN lesions or inhibition by GABAergic agonists developed impulsive-like behavior with deficit in response inhibition and motivational exacerbation (Baunez and Robbins, 1997; Phillips and Brown, 2000; Baunez et al., 2005). A study with manipulation using optogenetic techniques demonstrated that brief activation of the STN is sufficient to interrupt or pause behavior reenforcing its cognitive role in response suppression and stopping behavior (Fife et al., 2017). Moreover, it has been shown that deep brain stimulation (DBS) of the STN (STN-DBS), inducing inhibition of the STN, modulates reward-related behavior in rodents (Baunez et al., 2007; Baunez and Gubellini, 2010; Rouaud et al., 2010; Vachez and Creed, 2020). Similarly, injection of muscimol into the lateral portion of the sensorimotor territory in the dorsolateral STN of NHPs induced circling and atypical behavior mainly characterized by hypervigilance (Baron et al., 2002). Besides preclinical studies, clinical outcome also confirms this STN differential implication. STN-DBS widely used to improve motor symptoms in Parkinson’s disease (PD), also improves symptoms in refractory obsessive-compulsive disorder (OCD) (Mallet et al., 2002; Chabardes et al., 2020). Analysis of STN neuronal activity of parkinsonian patients showed an inhibition of beta oscillations (13-30 Hz) during movement, in the more dorsal territory of the STN (Morelli and Summers, 2023; van Wijk et al., 2023). Other studies have found cognitive behavioral involvement of the STN with an increase in alpha and theta oscillations in middle and ventral parts during decision-making tasks (Fumagalli et al., 2011; Bastin et al., 2014; Benis et al., 2020). Moreover, in parkinsonian patients, the STN limbic involvement has been illustrated by a decrease in alpha oscillations during reward-related processing (Kühn et al., 2005; Brücke et al., 2007; Eitan et al., 2013) and an increase in theta and gamma oscillations in the more medial part when receiving a reward (Rosa et al., 2013; Huebl et al., 2014). These results led to the investigation of the impact of theta-alpha band frequency stimulation on non-motor symptoms of Parkinson’s disease. Notably, Kelley and colleagues found that 4 Hz stimulation improved cognitive processes in parkinsonian patients (Kelley et al., 2018). Subsequent research provided further insights, showing that 10 Hz stimulation, in contrast to 130 Hz stimulation, decreased negative biases in parkinsonian patients, when applied intermittently in the right ventromedial STN (Mandali et al., 2020), and increased arousal and positive valence rating when applied bilaterally (Wang et al., 2023). These finding were supported by MRI and tracing studies of STN functional connectivity in NHPs and humans with healthy volunteers and parkinsonian patients: STN dorsal territory receives motor connections from the premotor cortex, supplementary motor area and primary motor cortex, and ventro-medial territory receives cognitive and limbic connections from the prefrontal, the orbitofrontal and anterior cortices (Levesque and Parent, 2005; Karachi et al., 2009; Lambert et al., 2012; Haynes and Haber, 2013; Plantinga et al., 2018; Petersen et al., 2019; Emmi et al., 2020). Based on those evidence, the tripartite model, dividing the STN into a motor, cognitive and limbic territory, has been proposed in primates but is still being discussed (Parent and Hazrati, 1995; Hamani et al., 2004, 2017; Mathai and Smith, 2011; van Wijk et al., 2020). Indeed, the number of territories, their sizes and their degree of segregation are still not well known (Keuken et al., 2012; Alkemade, 2013; Alkemade et al., 2015; Lambert et al., 2015), which remains a critical issue for patients undergoing STN-DBS. Non-motor effects have been reported in parkinsonian patients with hypomania when implanted in a more ventral position (Welter et al., 2014), or decline in verbal fluency when implanted in a more anterior location (Greif et al., 2021; Vos et al., 2021). Despite the recent development of segmented electrodes, designed to steer current in specific directions, some studies have questioned the clinical relevance of current steering and its potential clinical improvement of DBS (Ineichen et al., 2018; Abdollahifard et al., 2023). These results confirm the importance of electrode localization within the STN, and STN-DBS can still be responsible for unpredictable long-term side effects, depending on the location of active contacts into the STN (Rodriguez-Oroz et al., 2005; Frank et al., 2007; Temel et al., 2007; Hälbig et al., 2009; Okun et al., 2009; Bronstein et al., 2011; Zarzycki and Domitrz, 2020; Vinke et al., 2022; Kremer et al., 2023). A better understanding of the distribution of motor, cognitive, and limbic functions within the STN may help to further optimize electrode placement and thus the efficacy of STN-DBS. To address this issue, we investigated electrophysiological biomarkers through four contacts in the STN, using local field potential recordings from directional electrodes, in two healthy NHPs performing a task designed to specifically tease apart these three functions. We also studied the effects of directional stimulation of these contacts on the same behavioral components.

## Materials and methods

### Animals

This study was conducted with one male (M1) and one female (M2), 8 years old (*Macaca fascicularis*, CRP, Port Louis, Mauritius). They were pair housed in a temperature (22 ± 1°C) and humidity (50 ± 5%) controlled facility with a 12 h light–dark cycle. They had free access to primate chow and water, and supplemental fruits were given once a day. All procedures followed the European Communities Council Directive of 2010 (2010/63/UE) for care of laboratory animals with the recommendations of the French National Committee (2013/113) and were approved by the local Ethical Committee (#04; authorization n°2019013116115695).

### Behavioral task

**Figure 1:**
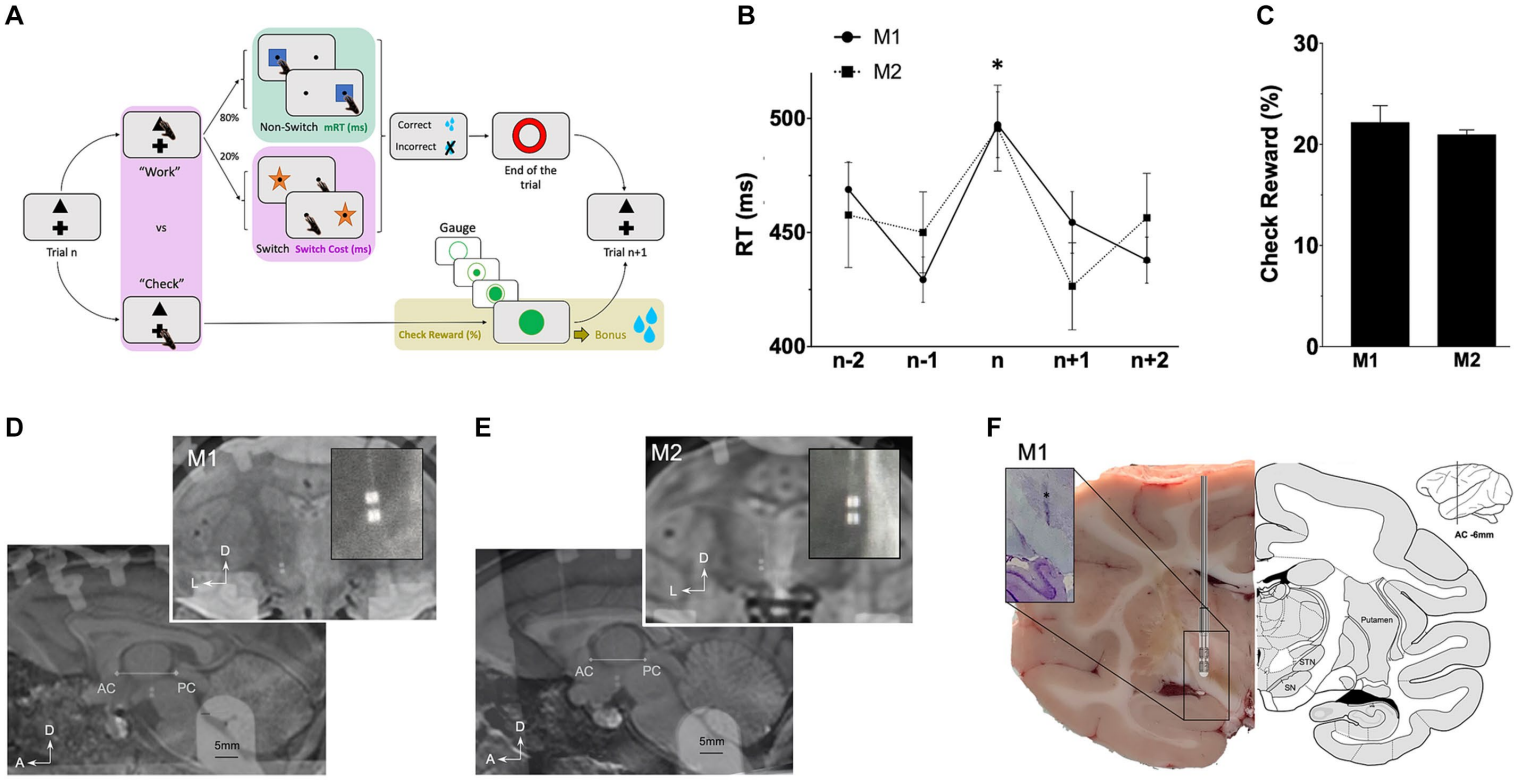
Behavioral task and leads location. **(A)** Rewarded task enabling the study of behavioral motor (green), cognitive (pink) and limbic (gold) components. On each trial, monkeys decided to “Work” on the main switching task, or “Check” the size of a gauge informing about the proximity of a bonus reward (see Methods). **(B)** Average response times (RT) in ms plotted against trial positions (n represents switch trials) ^*^p < 0.05, and **(C)** probabilities to check when the reward is available (check reward) expressed in percentage (%). **(D)** lead location for M1 on MRI scans on sagittal and coronal view **(E)** lead location for M2 on MRI scans on sagittal and coronal view. **(F)** Coronal section with electrode trace (*) for M1 after cresyl violet immunostaining (left) compared with atlas (right). A, anterior; AC, anterior commissure; D, dorsal; ic, internal capsule; L, lateral; M, medial; PC, posterior commissure; Put., putamen; SN, substantia nigra; SNC, pars compacta; SNR, pars reticulata; STN, subthalamic nucleus.

A manual counter demanding task, involving strategic decision-making, was adapted from prior studies (Everling and DeSouza, 2005; Isoda and Hikosaka, 2008; Stoll et al., 2016). This task specifically assessed motor, cognitive and limbic (reward-related) components of the behavior (Figure 1A). Monkeys were habituated to sit in a primate chair (Crist Instrument Co., MD, United States) in front of a touch screen (Elo Touch Solutions, Inc.). An open window allowed them to use their preferred hand (the right) to interact with stimuli presented on a grey background screen. At the beginning of each trial, monkeys had the choice to select either a triangle to “Work” and perform a switching task, or a cross to “Check” and see an increasing gauge informing of the proximity of a bonus reward. Those stimuli were always displayed at the same positions (at the upper center and at the bottom center respectively). The Work option consisted in a switching task with two different targets, a blue square (Non-Switch), or an orange star (Switch), which appeared in the periphery (randomly left or right side). Monkeys were required to touch the blue square while they had to touch on the opposite side of the orange star. Targets were randomly determined trial to trial, with a higher probability for Non-Switch than Switch (80–20%) to induce automatic behavior. In both cases, monkeys had to respond within 2000 ms and correct responses were rewarded (sweet liquid, 1 mL) by a computer-controlled system (Crist Instrument Co.). Then, a red circle appeared for 1,000 ms, indicated the End of the trial and the beginning of a new one with the Work or Check option. Instead of selecting the Work option, monkeys could choose to Check. A gauge was displayed for 5,000 ms, indicating the proximity of a bonus reward. Represented by a large green circle, the gauge was filled proportionally to the number of correct responses performed on the Work option (an inner green disk filled the large green circle). Incorrect responses did not affect the gauge. To avoid any anticipation, the number of correct responses required to fill the gauge was randomly selected from 8, 16, 24 or 32. To receive the bonus reward (sweet liquid, 5 mL), monkeys had to choose to Check when the gauge was full (same size of the inner green disk and the large green circle). This bonus reward remained available until the Check option was chosen, and once delivered, the gauge size was reset and a new random number was picked (8, 16, 24, 32).

### Behavioral analyses

Only sessions with more than four earned bonus rewards were included. Response times (RT), defined as the time between appearance of the stimulus and the touching response, were measured from correct trials only. They were termed motor RT (mRT, motor component of the task) and cognitive RT (cRT, cognitive component of the task) for non-switch (>1,700 trials, Supplementary Table S1) and switch (>300 trials, Supplementary Table S1) trials, respectively. Differences between all cRT and all mRT represented the Switch-Cost, i.e., time needed to adapt behavior to a change of rules and represent the cognitive component of the task (Supplementary Table S1; Figure 1B). Probabilities to Check when the gauge was full and the bonus reward was available were calculated in relation to the probabilities to Work and represent the limbic motivation to get the reward (Check Reward, >500 trials, Supplementary Table S1; Figure 1C).

### Surgery

After a training period of the task (5 days a week for 4 months), a directional lead was implanted. Surgery was performed under aseptic conditions and general anesthesia. Monkeys were first anesthetized with injection of ketamine (7 mg.kg^−1^) and xylazine (0.6 mg.kg^−1^), and, maintained under general anesthesia with isoflurane. Lidocaine 1% was used for local anesthesia. Saline solution (NaCl 0.9%, Sigma-Aldrich) was continuously infused intravenously during the operation for drug access and hydration. Analgesic/anti-inflammatory therapies (Ketoprofen 2 mg.kg^−1^) were delivered during the one-week post-operative period. Pre-operative 7 T MRI was acquired to identify the STN and per-operative radiographic X-rays allowed the localization of ventriculographic bi-commissural landmarks (anterior and posterior commissures -ACPC), obtained using a cannula stereotactically placed on the left lateral ventricular through which 2 mL of ventricular contrast (Iopamiron 200, iodine 200 mg/mL, Bracc) was injected. Micro-recordings (microelectrode of 250 μm diameter, 0.8–1.2MOhm, FHC) were used to locate borders of the STN, using specific firing rate and pattern. Right STN was implanted with segmented leads (Heraeus © - United States). Electrodes of 0.8 mm diameter consisted in four 0.5 mm contacts arranged in two rows (0.5 mm apart) and two columns. For both monkeys, the electrode was placed at the following stereotactic coordinates: anterior 6/12th of the ACPC line, 4–5 mm from the midline, with deepest contacts at the lower border of the STN. Segmented leads allowed directional recordings and stimulations of four contacts in the STN: dorsolateral (DL), dorsomedial (DM), ventrolateral (VL) and ventromedial (VM). The reference was fixed on the skull at the left occipital level. The implantation was verified by radiography of the final implantation merged to the pre-operative MRI (Figures 1D,E). A stainless-steel head holder (Crist Instruments, MD) was placed at the back of the skull to maintain monkey’s heads.

### Recordings analyses

Recordings began after a post-operative period of 2 weeks. Electrophysiological activity of four contacts in the STN (DL, DM, VL, VM) was recorded simultaneously using a common reference located at the occipital level using a multichannel system (AlphaOmega Engineering, Israel). Signals were sampled at 1375 Hz, clipped around specific events (±1,000 ms) and analyzed using MATLAB (The Mathworks, Natick MA, United States) and ImaGIN toolbox. Time-frequency representations of spectral power between 2 and 200 Hz were obtained using a multitaper sliding window. Orthogonal discrete prolate Slepian spheroidal (DPSS) tapers were applied in a frequency-dependent manner and according to the length of the sliding window. Single-trial power spectra were averaged for each valence condition and normalized on a pre-stimulus baseline interval (−1,000 to −250 msec) by calculating the base-10 logarithm of the ratio LdB (peristimulus power/baseline power (P/B)) and multiplying it by 10 to return decibel (dB) values: L_dB_ = 10 log_10_ (P/B). Power values are denoted as LdB power ± SEM. Specific frequency bands have been selected with band filters (theta, 4–8 Hz; alpha, 8–12 Hz; beta, 12–35 Hz; gamma, 35–200 Hz) and are represented as average power versus baseline over time (mean ± SEM).

### Directional stimulation

The opposing effects of continuous bipolar low frequency [LFS: 4 Hz, based on cognitive improvement with 4 Hz STN-DBS (Kelley et al., 2018)] and high frequency [HFS: 130 Hz, based on inhibition effect of STN-DBS in parkinsonian patients (Benabid et al., 2009)] stimulations were tested on four contacts in the STN (DL, DM, VL, VM). For each frequency and contact, ranges of stimulations were performed until side effects occurred (e.g., monocular and ipsilateral deviation, head rotation, lip contraction). Intensities were applied at 80% of the threshold of onset of side effects (from 0.08 mA to 0.2 mA), with a fixed width pulse (60 μs). Number of trials for each stimulated contacts at both frequencies are presented in Supplementary Table S2.

### Immunohistochemistry

M1 was deeply anesthetized with ketamine (10 mg/kg) and killed by an overdose of pentobarbital (25 mg/kg i.v.). The brain was removed from the skull and frozen using liquid nitrogen vapor before being stored at −80°C. To locate the electrode, 20 μm thick sections with its trace were mounted on silanized slides and marked with Cresyl violet. The sections were stained in a 1% cresyl violet solution (Sigma, C5042), dehydrated in baths of increasing concentrations of alcohol and then degreased in a xylene bath. M2 is still involved in a project, so immunochemistry could not be achieved.

### Statistical analyses

#### Behavioral data

Standard statistical methods using GraphPad Prism 8 were applied for all data comparisons (GraphPad Software Inc., United States). After testing the normal distribution of the data with a Shapiro test, one-way non-parametric ANOVA, Kruskal–Wallis tests, followed by Dunn’s multiple comparisons were applied for all behavioral data and comparison between ON and OFF stimulation condition (cRT, mRT, Switch-Cost, Check Reward). Statistical results for all trials across sessions are reported in Supplementary Tables S1, S2. Data are represented as mean ± SEM and a difference was considered statistically significant for a value of *p* < 0.05.

#### Statistical parametric maps

*Post-hoc t*-tests for time-frequency representations were Bonferroni corrected, with “one-sample” *t*-test to compare signal of one event, and “two-sample” *t*-test allowed the discrimination of significant differences between two events (*T*-values). Those analyses were plotted as statistical parametric maps (SPM) with significant statistical value of *p* < 0.05. When a cluster of interest was found on SPM, Shapiro’s test was used to test normality and repeated-measures analysis of variance (ANOVA) followed by Tukey’s test were performed using average power changes within the identified windows and frequency band, i.e., at the presentation of the motor cue and the beginning of the movement compared to a resting state (motor SPM), around the touch to either choose to work compared to check and, response to a Switch compared to a Non-Switch trials (cognitive SPMs), at the significant changes for the reward delivery with the full gauge compared to the gauge being filled (emotive SPM) (Relative power, L_dB_). Statistical results are reported in Supplementary Table S3. Data are represented as mean ± SEM and a difference was considered statistically significant for a value of *p* < 0.05.

## Results

### General behavior

Motor response times (mRT) on Non-Switch trials were similar for both monkeys (447.9 ± 2.1 ms for M1; 445.6 ± 3.1 ms for M2). Switching from automatic to controlled behavior led to significant longer cognitive response times (cRT) than mRT with a similar switch-cost for all monkeys (cRT-mRT = 51.4 ± 5.0 ms for M1 *p* < 0.0001; 45.1 ± 7.4 ms for M2 *p* < 0.0001; Kruskal–Wallis) (Supplementary Table S1; Figure 1B). Probabilities to check the gauge when the reward bonus was available, representing motivation to get the reward, were similar for M1 and M2 (19.8 ± 0.1% for M1; 22.0 ± 0.2% for M2) (Figure 1C).

### Recordings and stimulations

Segmented leads for NHPs allowed recordings and stimulation of four contacts in the STN: dorsolateral (DL), dorsomedial (DM), ventrolateral (VL) and ventromedial (VM). Low frequency (LFS) and high frequency (HFS) stimulations were applied during the task and intensities were previously determined with ranges of stimulations for each contact in both frequencies. Typical side effects (e.g., monocular and ipsilateral deviation with medial contacts and head rotation or lip contraction with lateral contacts) confirmed the lead position verified with MRI merged with implantation scans (Figures 1D,E). Finally, for M1, the precise location of the electrode was confirmed postmortem showing implantation in the STN (Figure 1F).

### Motor component

**Figure 2:**
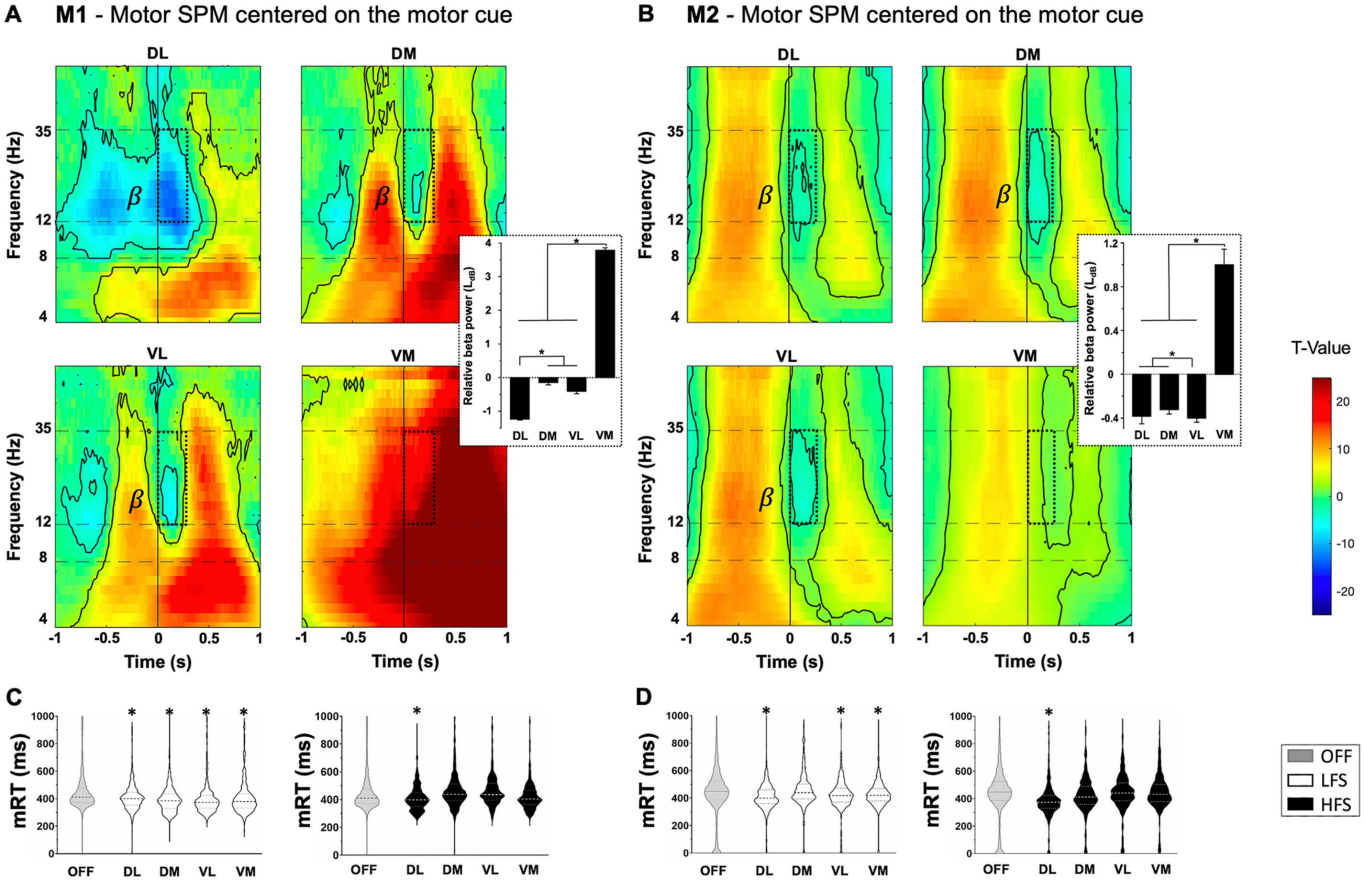
Motor component with statistical parametric maps (SPM) of the activity of subthalamic contacts for M1 **(A)** and M2 **(B)**, centered on the appearance of non-switch stimulus (vertical line at 0) relative to a resting state (*T*-value). Beta frequency range (*β*) decreased during movement (dotted windows) with beta power values displayed on the right, expressed in LdB and mean ± SEM. Significant values are encircled with solid lines *p* < 0.05. **(C)** M1 and **(D)** M2 representation of the distribution of motor response times (mRT) in milliseconds (ms) with low (LFS, white) and high (HFS, black) frequency stimulations, compared to OFF-stimulation condition (OFF, gray), for each contact in the subthalamic nucleus. The large dotted lines represent the median and small dotted lines represent the quartiles. ^*^p < 0.05. DL, dorsolateral; DM, dorsomedial; VL, ventrolateral; VM, ventromedial.

For technical and ethical reasons, ipsilateral STN activity were recorded during the execution of the behavioral task with the right hand. For M1 and M2, beta power (12-35 Hz) significantly decreased at the onset of Non-Switch stimulus corresponding to the motor component to touch the screen, and increased after the movement (*p* < 0.05, one-sample *t*-test) (Figures 2A,B). DL contacts were more impacted by the beta power decrease than VM ones (M1: −1.2 ± 0.03LdB for DL vs. 3.8 ± 0.08LdB for VM, *p* < 0.0001; M2: −0.38 ± 0.07LdB for DL vs. 1.0 ± 0.14LdB for VM, *p* < 0.0001, ANOVA). Specifically applied on DL contacts, HFS induced significantly faster automatic mRT compared to the OFF-stimulation condition (−37.9 ± 5.3 ms for M1; −60.5 ± 6.6 ms for M2, Kruskal–Wallis) (Figures 2C,D). LFS induced significantly faster mRT compared to OFF-stimulation condition for all contacts, except DM for M2 (M1: −38.0 ± 4.8 ms for DL; −51.2 ± 5.2 ms for DM; −54.9 ± 5.8 ms for VL; −40.2 ± 6.6 ms for VM, *p* < 0.0001; M2: −35.5 ± 5.7 for DL; −22.5 ± 6.2 for VL; −17.2 ± 5.5 for VM, *p* < 0.0001, Kruskal–Wallis) (Figures 2C,D). The overall success was not impacted by stimulations (Supplementary Table S2).

### Cognitive component

**Figure 3:**
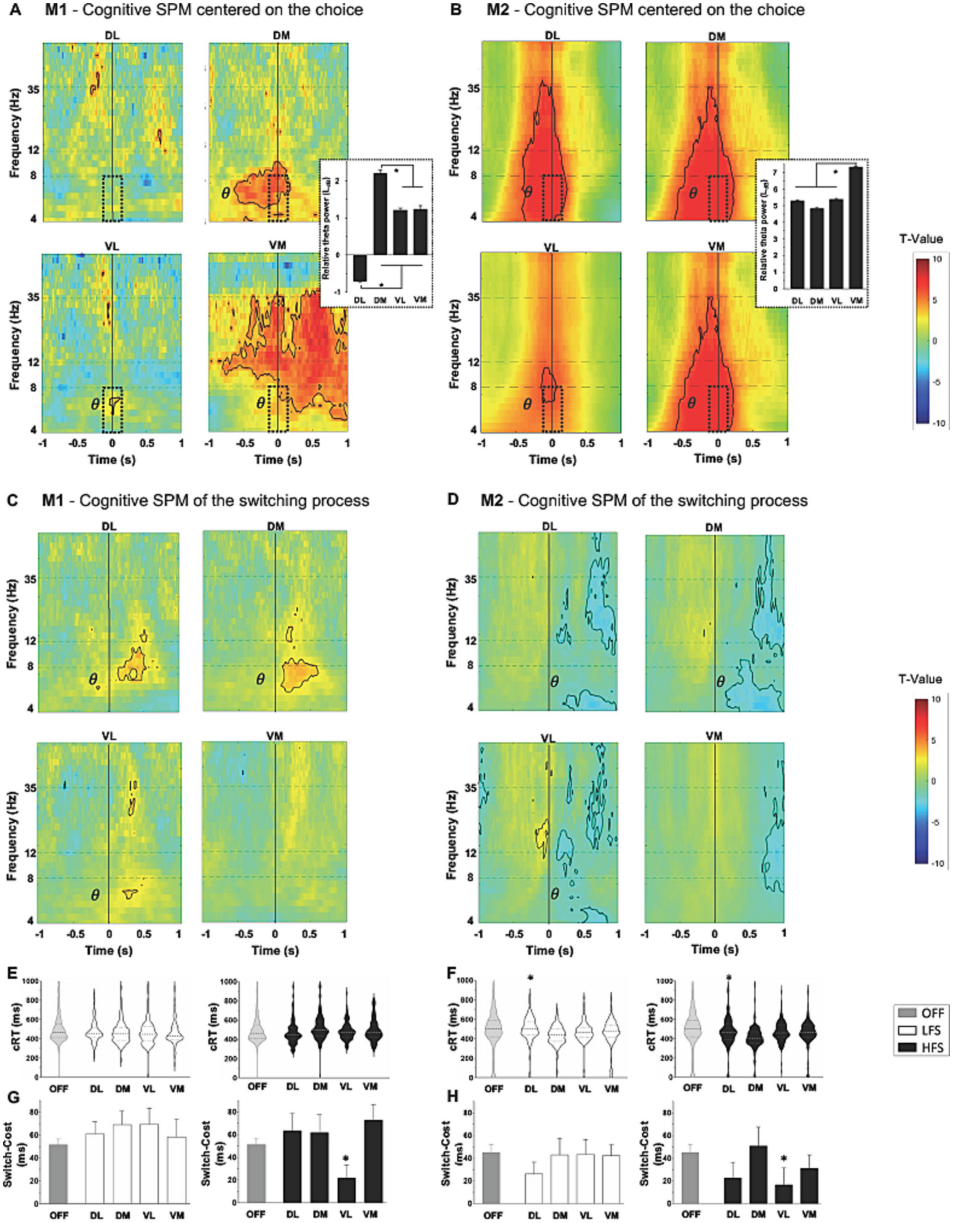
Cognitive component with statistical parametric maps (SPM) of the activity of subthalamic contacts, centered on the cognitive to work relative to check, and on the switch relative to non-switch trials (vertical line at 0, *T*-values), for M1 [**(A,C)** respectively] and for M2 [**(B,D)** respectively]. An increase in the theta frequency range (*θ*) is observed during the choice (dotted windows) with theta power values displayed on the right, expressed in LdB and mean ± SEM. Opposite theta changes are observed on switch compared to non-switch trials between M1 and M2. Significant values are encircled with solid lines *p* < 0.005. **(E)** M1 and **(F)** M2 representation of the distribution of cognitive response time (cRT) and, **(G)** M1 and **(H)** M2 switch-cost in milliseconds (ms) expressed in mean ± SEM; with low (LFS, white) and high (HFS, black) frequencies stimulation, compared to OFF-stimulation condition (OFF, gray), for each contact in the subthalamic nucleus. The large dotted lines represent the median and small dotted lines represent the quartiles. ^*^p < 0.05. DL, dorsolateral; DM, dorsomedial; VL, ventrolateral; VM, ventromedial.

When monkeys chose to Work rather than Check, a significant increase in theta band (4–8 Hz) was observed around the decision time (at 0) in all contacts, except DL for M1 (*p* < 0.05, two-sample *t*-test) (Figures 3A,B). This cognitive marker was more present in medial contacts, DM for M1 and VM for M2 (M1: 2.2 ± 0.08LdB for DM vs. 1.2 ± 0.06LdB for VL, *p* < 0.0001; M2: 7.3 ± 0.05LdB for VM vs. 5.3 ± 0.6LdB for DL, *p* < 0.0001, ANOVA). Opposite theta oscillations were observed when comparing the response to Switch and Non-Switch trials for the animals. For M1, an increase in theta band was observed in all contacts except VM, while a decrease in the same band was observed in all contacts but VM for M2 (*p* < 0.05, two-sample *t*-test) (Figures 3C,D). Overall cRT was not impacted by stimulations, but a decrease was observed with both LFS and HFS when applied in DL for M2 (436.2 ± 9.43 ms at LFS, *p* = 0.0002 and 407.7 ± 12.61 ms at HFS, *p* < 0.0001, vs. 490.7 ± 6.67 ms OFF-stimulation, Kruskal–Wallis) (Figures 3E,F). Switch-Cost decreased for both monkeys when HFS was applied in VL contacts, compared to OFF-stimulation condition (−29.5 ± 12.5 ms for M1, *p* = 0.0003; −28.0 ± 16.4 ms for M2, *p* = 0.0006, Kruskal–Wallis) (Figures 3G,H). The overall success was not impacted by stimulations (Supplementary Table S2).

### Reward-related component

**Figure 4:**
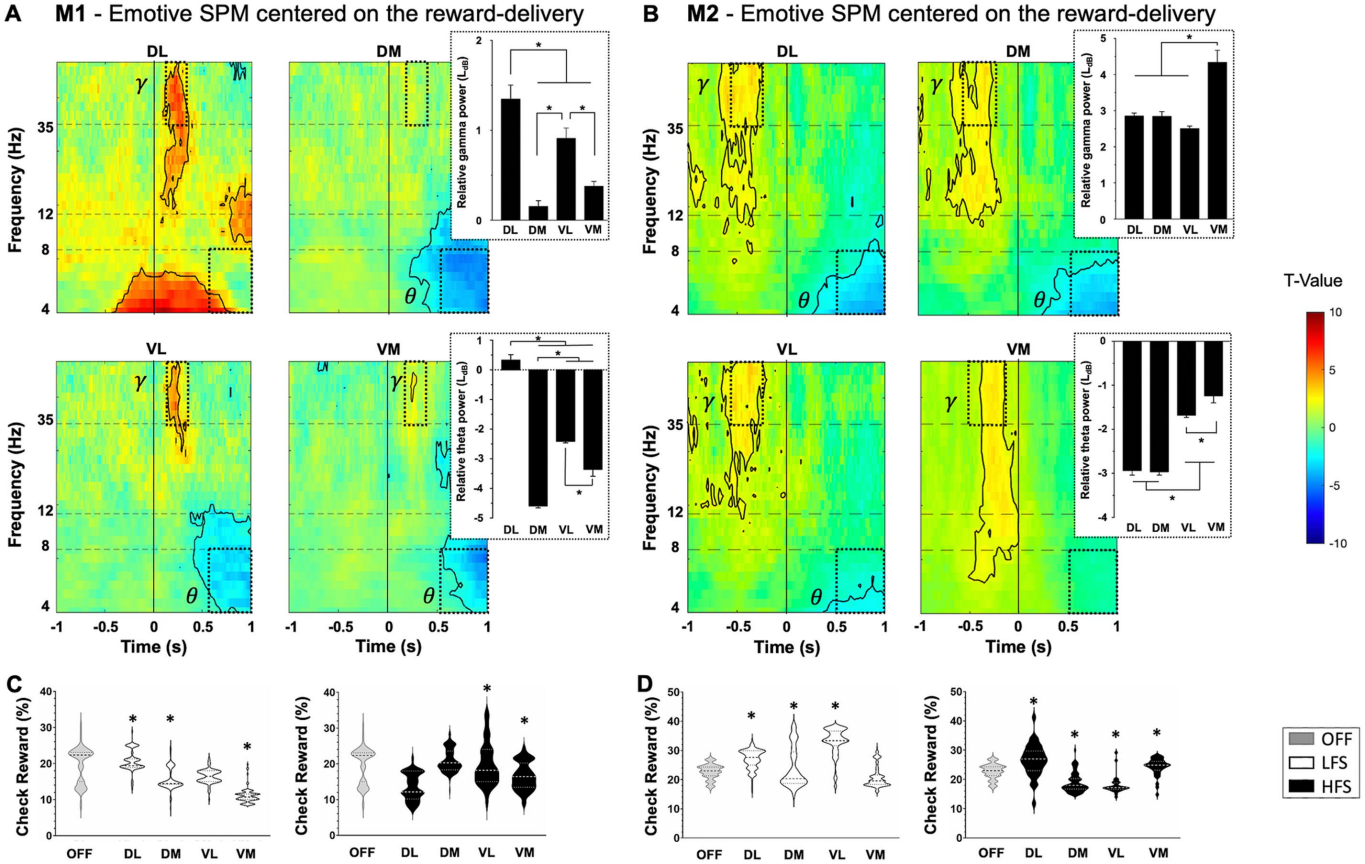
Reward-relative component with statistical parametric maps (SPM) of the activity of subthalamic contacts for M1 **(A)** and M2 **(B)**, centered on the full gauge with reward delivery (vertical line at 0) relative to the gauge view without the reward (*T*-values). An increase in gamma frequency range (*γ*) followed by a decrease in theta frequency range (*θ*) are observed when receiving the reward (dotted windows) with theta and gamma power values displayed on the right, expressed in LdB and mean ± SEM. Significant values are encircled with solid lines *p* < 0.005. **(C)** M1 and **(D)** M2 representation of the distribution of probability to check when the bonus reward is available (check reward), in percentage (%) with low (LFS, white) and high (HFS, black) frequency stimulations, compared to OFF-stimulation condition (OFF, gray), for each contact in the subthalamic nucleus. The large dotted lines represent the median and small dotted lines represent the quartiles. Values are expressed in mean ± SEM and ^*^p < 0.05. DL, dorsolateral; DM, dorsomedial; VL, ventrolateral; VM, ventromedial.

For both monkeys, a significant increase in gamma oscillations (>35 Hz) followed by a significant decrease in theta oscillations were observed when the reward bonus was delivered (*p* < 0.05, two-sample *t*-test) (Figures 4A,B). This pattern was found in all contacts, except DL for M1. However, the timing was slightly different between monkeys, with an increase in gamma oscillations during the reward delivery for M1, whereas it appeared before the reward was obtained for M2. Stimulation modulated differently the motivation to get the reward in M1 and M2. However, for both monkeys, HFS applied in VL contacts decreased the check for the bonus reward compared to OFF stimulation (−6.8 ± 0.3% for M1, *p* < 0.0001; −3.7 ± 0.4% for M2, *p* < 0.0001, Kruskal–Wallis) (Figures 4C,D).

## Discussion

In this study, we characterized markers within the STN by using directional LFP recordings and stimulations. Beta oscillations, mainly observed in the dorsolateral STN, were associated with motor component of behavior; theta oscillations, more present in the medioventral STN, were associated with cognitive processes, and gamma-theta oscillations were found throughout the STN, correlating with the reward-related limbic component. Furthermore, our study demonstrated that STN-HFS improved motor reaction times when applied in the dorsolateral part, as well as the STN-LFS, applied throughout the STN. Only STN-HFS applied in the ventrolateral part modified cognitive performances. Finally, both HFS and LFS, when applied globally to the whole STN, were able to modify reward-related limbic behavior. These findings were observed in healthy macaques and provide novel information to understand the effect of STN-DBS, considering both frequency and stimulated contact, which is a critical research goal for clinical application.

### Motor component

The STN involvement in motor behavior have already been described in rodents (Dybdal and Gale, 2000) and NHPs (Hamada and DeLong, 1992; Karachi et al., 2005). Moreover, other studies with parkinsonian patients have shown a decrease in beta band activity during movement, followed by an increase known as rebound (Alegre et al., 2013; Zavala et al., 2013; Pötter-Nerger et al., 2017; Alhourani et al., 2020). This can be considered as electrophysiological markers of motor behavior for planification and execution and was found throughout the STN but more present in the DL contact compared to VM, in agreement with the dorsolateral-ventromedial gradient hypothesis (Hamani et al., 2004; Mallet et al., 2007). Furthermore, mRT was improved, by HFS in DL contacts for both monkeys. The hyperdirect pathway, which anatomically connects somatosensory and motor cortices to the dorsal STN territory supports this finding (Litvak et al., 2012; Haynes and Haber, 2013; Fischer et al., 2017; Alhourani et al., 2020). LFS had the same effect regardless of the contact stimulated. Interestingly, low frequency coherence was found in the top-down functional coupling of the hyperdirect pathway between cortex and STN, in NHP and parkinsonian patients (Haynes and Haber, 2013; Alkemade et al., 2015). In physiological context, LFS may amplify this connection, leading to faster mRT. However, this improvement may reflect cognitive processes related to impulsivity, known to be modulated with LFS (Kelley et al., 2018), but the lack of increase in error rate and ease of the task cannot lead to conclusions. Moreover, it should be emphasized that right STN activity was recorded while using the ipsilateral limb (right) to perform the task. Indeed, technical reasons linked to the set-up of our operating room and ethical and animal welfare reasons related to a manual preference for the right hand explain the recordings made on the ipsilateral side. Numerous studies in NHPs have shown that the primary motor cortex and basal ganglia are involved in the control of movements predominantly on the contralateral side of the body (Matsumura et al., 1992; Dum and Strick, 1996; Lacroix et al., 2004; Rosenzweig et al., 2009; Polyakova et al., 2020). However, several subsequent studies highlight M1 activity in relation to ipsilateral and contralateral movements in NHP and humans (Donchin et al., 1998; Cisek et al., 2003; Diedrichsen et al., 2013; Bundy and Leuthardt, 2019; Heming et al., 2019; Gardner et al., 2022). Heming and colleagues found that contralateral responses tended to be larger and earlier than ipsilateral responses, and that disruption activity in M1 related to the ipsilateral limb also reflected features related to motor output in rhesus macaques (Heming et al., 2019). Similarly, Gardner and colleagues observed a comparable, but weaker and shorter, response between ipsi- and contralateral M1 during hand reaching and grasping movements in NHP (Gardner et al., 2022). Furthermore, a study in rats recorded changes in ipsi- and contralateral STN during ipsilateral microinjection of excitatory or inhibitory drugs into the parafascicular nucleus, highlighting the importance of this thalamic structure in the bilateral regulation of basal ganglia activity (Mouroux et al., 1995). Finally, Alegre and colleagues found bilateral beta changes in the human STN during movements of either hand, suggesting that movement-related activity in the STN has, by and large, a bilateral representation and probably reflects cortical input (Alegre et al., 2005). Overall, these studies consolidate the results obtained in the STN with ipsilateral limb movements, although it would be interesting to carry out recordings of the contralateral STN, which should show a stronger and longer motor response and would also provide further temporal knowledge of these bilateral movement-related activity.

### Cognitive component

Theta oscillations throughout the STN along a medioventral-laterodorsal gradient were recorded when monkeys had to choose between Work or Check. This increase in theta oscillations appeared mainly during the decision processes and before the decision action of touching the stimulus, in the DM contact for M1 and in all contacts except the VL for M2. For both monkeys, this marker appeared during the decision action in the VL. This event can be considered as electrophysiological marker of decision-making processes in this task. Other studies have shown similar changes in theta activity, which could be a physiological marker of the conflict state in parkinsonian patients (Cavanagh et al., 2011; Alegre et al., 2013; Herz et al., 2016). Isoda and Hikosaka showed that cognitive processes for switching from automatic to adapted behavior implicated “switch neurons” in the STN of NHP, which were mainly located in the ventral territory (Isoda and Hikosaka, 2007, 2008). It has been proposed that direct anatomical connections between prefrontal cortex and ventral STN would facilitate a more deliberative response process to choose the most appropriate behavior during a conflict state (Miller, 2000; Cavanagh et al., 2011; Alegre et al., 2013; Haynes and Haber, 2013). In line with these previous studies, a change in theta oscillations was observed during cognitive processes when comparing responses on Switch and Non-Switch trials. However, animals showed opposite changes. Indeed, M1 presented an increase in theta oscillations, representing conflict processes and cognitive control, as previously described in the literature (Zavala et al., 2013, 2018). M2, on the other hand, presented a decrease in theta oscillations that could be due to the small number of trials with switching compared to the number of trials without switching, to a learning phenomenon, or to the simplicity of the task after this many trials. We also showed that cognitive Switch-Cost was decreased with HFS in VL contacts for both monkeys, demonstrating improved cognitive performance. We hypothesized that HFS could inhibit the STN, bypassing its “break” role and making faster cRT even with a switching rule. No significant effect was observed with LFS, while an improvement in cognitive performance was expected. This was recently demonstrated with 4 Hz stimulation of STN dorsal contacts, consistent with the hyperdirect pathway in cognitive control, which improved cognitive deficits in parkinsonian patients (Kelley et al., 2018).

### Reward-related component

The STN role in the behavioral limbic component was investigated using single unit recordings in rodents (Baunez et al., 2002; Lardeux et al., 2009) and NHPs (Darbaky et al., 2005; Espinosa-Parrilla et al., 2013; Nougaret et al., 2022), with distinction between reward delivery, reward expectation and integration of the motivational value of the stimulus. Interestingly, these studies revealed motivation-responsive neuronal activities throughout the STN, without specific localization. This is in line with our study demonstrating changes, early in gamma and followed by theta frequency bands associated with the delivery of the reward bonus, throughout the STN and not in specific contacts. Early gamma band response was also found in the STN of Dopa-treated parkinsonian patients and may represent local encoding of increased attention, which varies with stimulus arousal (Huebl et al., 2014). Early gamma activity could increase limbic attention as a reward approaches, representing the reward expectation, as observed for M2. The amygdala has been shown to be involved in stimulus arousal in studies with amygdala-lesioned patients (Bechara et al., 1995; Gläscher and Adolphs, 2003) and is associated with emotional intensity, regardless of stimulus valence (Lin et al., 2020). Thus, the role of the STN in limbic attention could be driven by its functional connection from the amygdala, either directly (Shi et al., 2007; Péron et al., 2015) or indirectly through ventral striatum, putamen, and ventral pallidum (Amaral and Insausti, 1992), explaining the lack of clear gradient in the STN. Changes in theta band activity during the reward delivery have also been found throughout the STN in rodents (Degoulet et al., 2021) and patients with PD (Benis et al., 2020), pathological gambling (Rosa et al., 2013) or OCD (Bastin et al., 2014). Other studies showed theta oscillations associated with limbic processing in ventrolateral STN, in parkinsonian patients (Rappel et al., 2020) and in Dopa-treated parkinsonian patients with impulse control disorder (Rodriguez-Oroz et al., 2011). These results are coherent with the gamma and theta oscillations associated to limbic processes found in our study. However, an early increase in gamma oscillations was observed for M2, suggesting a role for this marker in the reward expectation, whereas it appeared later for M1, during the reward delivery. This distinction between monkeys is part of the inter-individual variability underlying the reward-related behaviors, involving the cortico-subcortical connection with the STN, and influenced by the internal state of the individual (Paulus, 2007; Keramati and Gutkin, 2014; Morris et al., 2017; Lewis et al., 2021). Furthermore, we showed that LFS and HFS modulate motivation to check for the bonus reward in a heterogeneous manner, depending on the frequency, stimulated contact, and monkey. Since we hypothesized a diffuse limbic territory in the STN, it is possible that behavioral impacts differ among stimulated neurons, depending on contact and frequency. Similarly, STN-DBS can lead to various limbic side effects such as apathy, depression, or mania in PD, specifically with more ventral stimulations (Houeto et al., 2002; Temel et al., 2006; Krack et al., 2010), but can improve limbic symptoms of OCD (Mulders et al., 2017; Voon et al., 2017).

### HFS versus LFS

Although underlying mechanisms of DBS remain unclear, different hypotheses have been proposed to explain the effect of HFS and the most common is known as “information lesion” in stimulated neurons. Indeed, HFS is thought to induce action potentials that suppress the transmission of low-frequency signals, overriding other intrinsic activities of stimulated neurons, thus, limiting the propagation of activity throughout the network (Grill et al., 2004; Lozano et al., 2019). HFS at 130 Hz improves behavioral symptoms such as motor impairment in PD or cognitive/limbic impairment in OCD but, for unclear reasons, may also induce motor, cognitive and limbic side effects (Houeto et al., 2002; Temel et al., 2006; Mulders et al., 2017). Therefore, recent studies have tried alternative frequencies, such as 4 Hz, leading to improved cognitive performance in parkinsonian patients (Kelley et al., 2018). Here, we did not replicate this effect. Since cognitive processes regroup a set of mechanisms, including executive functions, attention, or switching for example, the difference in the cognitive processes studied could explain the difference in results. Another hypothesis may be inherent to the healthy compared to the pathological state. Behavioral modulation by stimulation may be different depending on the affected components in pathologies. Further studies, in a pathological context, using directional DBS lead and this behavioral task, could reinforce the specific behavioral effects of HFS or LFS.

### Functional organization

**Figure 5:**
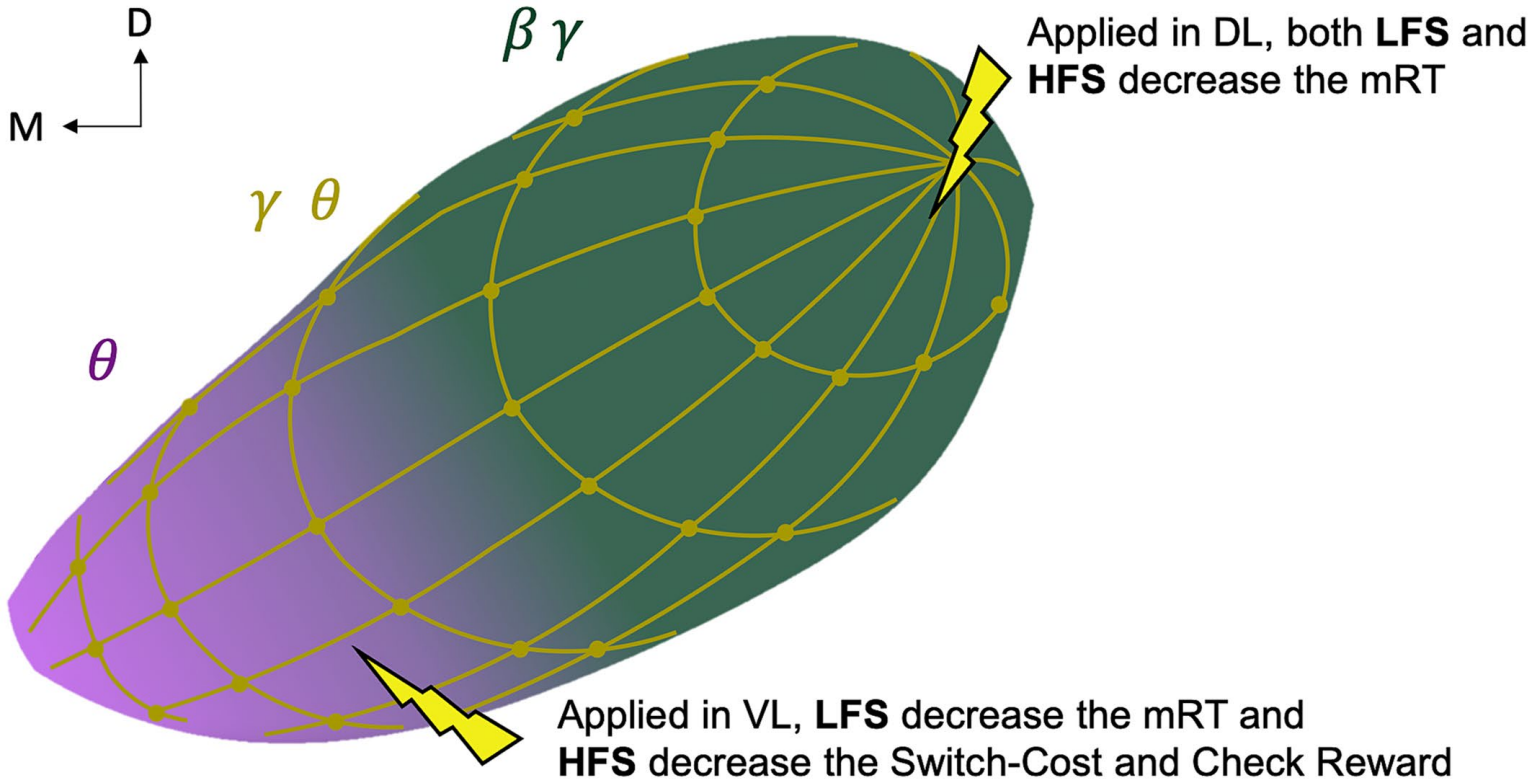
Schematic representation of the study-based bipartite functional organization of the subthalamic nucleus. Representation of the oscillations with motor beta-gamma (green, *β* *γ*) and cognitive theta (pink, *θ*) gradients and a diffuse reward-related theta-gamma (gold, *θ* *γ*) distribution; and representation of the behavioral effect of directional low (4 Hz, LFS) and high (130 Hz, HFS) stimulation. D, dorsal; M, medial; L, lateral; V, ventral; mRT, motor response time.

Even if the recordings did not cover the entire nucleus and were performed in the ipsilateral STN of the limb who performed the task, our results suggest an overlapping bipartite model of functional STN with a more dorsolateral motor territory, consistent with the classical tripartite model (Alexander et al., 1986; Albin et al., 1989; Parent and Hazrati, 1995; Nambu et al., 2002; Hamani et al., 2004), supporting a dorsolateral-ventromedial gradient (Mallet et al., 2007), and, a more ventral cognitive territory as a brake during decision conflict (Alexander et al., 1986; Parent and Hazrati, 1995; Hamani et al., 2004), supporting a ventromedial-dorsolateral gradient. However, unlike the classic tripartite model, we found a diffuse distribution of the reward-related marker reflecting limbic function in the whole STN (Figure 5).

### Limitation of our study

The localization of electrodes could be supplemented by histological analysis for M2, which could provide more accurate information on the exact position of the contacts and a better comparison between the animals. However, intraoperative single unit micro-recordings and ventriculography co-registered with pre-operative MRI, allowed us to validate the position and orientation in the STN. Moreover, the specific type of stimulation direction-related side effects observed during the stimulation ranges of each contact were also consistent with the electrode orientation (e.g., monocular, and ipsilateral deviation, head rotation or lip contraction when HFS was applied in the lateral contacts). However, the differences in the electrophysiological recordings between the animals could be explained by a different location of the electrode in the STN. We assume that the electrodes were not located exactly at the same place within the STN with a more posterior position for M1 and a more medial position for M2, because such surgery is never replicable to perfection (differences of implantation are also found in human), and because M1 and M2 are not of the same sex. Indeed, the sexual dimorphism present in the macaque at morphological level (Ravosa, 1991) induces a smaller size in the female (M2) with a smaller STN and therefore different recorded territories from M1 (AC-PC in M1: 11.2 mm; AC-PC in M2: 9.2 mm). Nevertheless, the more medial location of the electrode for M2 may explain why we observed less beta desynchronization for the motor component in DL for M2 compared with M1 and more theta oscillations for the cognitive component in the lateral contacts for M2 compared with M1. Finally, directional stimulation induced similar results between the two animals, except for the motivation to check the reward. Disparate effects were observed between M1 and M2 as a function of stimulated contact and frequency. This could reinforce the STN organization with a more lateral involvement in the motor component, a more medial involvement in the cognitive component and a global involvement of the STN in the limbic component. The use of single unit recordings could provide better spatial resolution of the STN activity correlated with motor and cognitive components.

## Data availability statement

The original contributions presented in the study are included in the article/Supplementary material, further inquiries can be directed to the corresponding author.

## Ethics statement

The animal study was approved by the local ethical committee (#04; authorization n°2019013116115695). The study was conducted in accordance with the local legislation and institutional requirements.

## Author contributions

MB: Conceptualization, Data curation, Formal analysis, Investigation, Methodology, Writing – original draft. SC: Writing – review & editing. VF: Methodology, Writing – review & editing. EP: Methodology, Writing – review & editing. JB: Writing – review & editing. BP: Conceptualization, Funding acquisition, Methodology, Supervision, Writing – review & editing.
