# Supplementary material for: Contribution of the subthalamic nucleus to motor, cognitive and limbic processes: an electrophysiological and stimulation study in monkeys
Source: Front Neurosci. 2024 Feb 22;18:1257579. doi: 10.3389/fnins.2024.1257579 (PMC10918855; doi:10.3389/fnins.2024.1257579)
Supplement: Supplementary file 1 [file Table_3.pdf]

frontiers

Supplementary Material

Supplementary Figures and Tables

	Trials mRT	mRT (ms) mean ± SEM	Trials cRT	cRT (ms) mean ± SEM	p-value	Switch-Cost (ms) mean ± SEM	Trials Check Reward	Check Reward (%) mean ± SEM
**M1**	3639	447.9 ± 2.11	943	499.3 ± 4.48	<0.0001	51.4 ± 4.95	1182	19.8 ± 0.12
**M2**	2973	445.6 ± 3.14	740	490.7 ± 6.67	<0.0001	45.1 ± 7.37	327	22.02 ± 0.23

Supplementary Table 1: Behavioral performances on the task for M1 and M2 for all trials across all sessions.

		Trials mRT	mRT (ms) mean ± SEM	p-value OFF/ON	Trials cRT	cRT (ms) mean ± SEM	p-value OFF/ON	Switch-Cost (ms) mean ± SEM	p-value OFF/ON	Total number of trials	Proba Correct (%) mean ± SEM	p-value OFF/ON	Trials Check Reward	Check Reward (%) mean ± SEM	p-value OFF/ON
**M1**	OFF	3639	447.9 ± 2.11	-	943	499.3 ± 4.48	-	51.4 ± 4.95	-	4582	97.4 ± 0.39	-	1182	19.8 ± 0.12	-
	DL	503	409.9 ± 4.36	<0.0001	124	470.7 ± 9.88	>0.9999	60.8 ± 10.80	>0.9999	627	95.7 ± 0.94	>0.9999	174	15.52 ± 0.27	<0.0001
LFS	DM	465	396.7 ± 4.77	<0.0001	112	465.6 ± 10.81	>0.9999	68.9 ± 11.81	>0.9999	577	97.4 ± 0.80	>0.9999	147	16.33 ± 0.30	<0.0001
(4Hz)	VL	384	393.0 ± 5.45	<0.0001	99	462.4 ± 13.07	>0.9999	69.4 ± 14.16	>0.9999	483	96.3 ± 0.76	>0.9999	105	20.95 ± 0.40	0.0755
	VM	382	407.7 ± 6.23	<0.0001	90	466.1 ± 14.41	>0.9999	58.4 ± 15.70	>0.9999	472	95.3 ± 0.77	>0.9999	159	10.69 ± 0.25	<0.0001
	DL	342	410.0 ± 4.84	0.0002	77	473.4 ± 14.44	>0.9999	63.4 ± 15.23	>0.9999	419	97.4 ± 0.44	>0.9999	87	20.69 ± 0.43	0.4755
HFS	DM	382	464.0 ± 6.25	0.0004	99	525.7 ± 14.5	>0.9999	61.7 ± 15.79	>0.9999	481	98.6 ± 0.37	>0.9999	118	19.49 ± 0.36	0.9961
(130Hz)	VL	364	464.6 ± 5.87	<0.0001	97	486.5 ± 9.87	>0.9999	21.9 ± 11.49	0.0003	461	97.5 ± 0.84	>0.9999	154	12.99 ± 0.27	<0.0001
	VM	340	424.3 ± 5.32	>0.9999	89	497.1 ± 12.54	>0.9999	72.8 ± 13.62	>0.9999	429	98.1 ± 0.47	>0.9999	111	16.22 ± 0.35	<0.0001
**M2**	OFF	2973	445.6 ± 3.14	-	740	490.7 ± 6.67	-	45.1 ± 7.37	-	3713	92.6 ± 0.73		327	22.02 ± 0.23	-
	DL	414	410.1 ± 4.81	<0.0001	102	436.2 ± 9.43	0.0002	26.1 ± 10.58	>0.9999	516	97.3 ± 0.68	0.0406	86	27.91 ± 0.48	<0.0001
LFS	DM	351	457.9 ± 6.61	>0.9999	101	500.7 ± 13.28	>0.9999	42.8 ± 14.83	>0.9999	452	93.0 ± 1.69	>0.9999	83	24.09 ± 0.47	0.0019
(4Hz)	VL	424	423.1 ± 5.43	<0.0001	116	466.3 ± 12.29	>0.9999	43.2 ± 13.40	>0.9999	540	92.8 ± 0.84	>0.9999	72	33.33 ± 0.56	<0.0001
	VM	534	428.4 ± 4.47	<0.0001	137	470.6 ± 8.56	>0.9999	42.2 ± 9.65	>0.9999	671	95.4 ± 0.81	>0.9999	149	20.81 ± 0.33	0.0827
	DL	425	385.1 ± 5.85	<0.0001	106	407.7 ± 12.61	<0.0001	50.9 ± 16.74	>0.9999	531	95.0 ± 0.96	>0.9999	91	26.37 ± 0.46	<0.0001
HFS	DM	413	422.3 ± 6.69	<0.0001	101	473.2 ± 15.35	>0.9999	22.6 ± 13.90	>0.9999	514	92.4 ± 1.99	>0.9999	114	18.42 ± 0.36	<0.0001
(130Hz)	VL	435	441.1 ± 6.89	>0.9999	122	460.8 ± 12.94	>0.9999	16.7 ± 14.66	0.0006	557	93.6 ± 0.95	>0.9999	131	18.32 ± 0.33	<0.0001
	VM	506	441.9 ± 6.01	>0.9999	126	472.9 ± 10.16	>0.9999	31.0 ± 11.80	>0.9999	632	92.0 ± 0.91	>0.9999	105	24.76 ± 0.42	<0.0001

Supplementary Table 2: Behavioral performances on the task for M1 and M2, for all trials with stimulation of the subthalamic nucleus. Macroelectrode contacts are indicted as follows: DL=dorsolateral, DL=dorsomedial, VL=ventrolateral, VM=ventromedial

		DL		DM		VL		VM			DL-DM	DL-VL	DL-VM	DM-VL	DM-VM	VL-VM
		mean	SEM	mean	SEM	mean	SEM	mean	SEM	n	p-value	p-value	p-value	p-value	p-value	p-value
**M1**	Limbic theta	0.33	0.18	-4.59	0.07	-2.41	0.05	-3.36	0.23	2013	<0.0001	0.02	<0.0001	<0.0001	0.44	<0.0001
	Limbic gamma	1.35	0.16	0.15	0.06	0.91	0.12	0.38	0.05	2013	<0.0001	<0.0001	<0.0001	<0.0001	<0.0001	<0.0001
	Cognitive theta	-0.70	0.02	2.19	0.08	1.21	0.05	1.23	0.09	943	<0.0001	<0.0001	<0.0001	<0.0001	<0.0001	0.99
	Motor beta	-1.24	0.03	-0.15	0.07	-0.41	0.07	3.79	0.08	3992	<0.0001	<0.0001	<0.0001	0.17	<0.0001	<0.0001
**M2**	Limbic theta	-2.94	0.10	-2.96	0.08	-1.68	0.05	-1.24	0.16	`1082	1.00	<0.0001	<0.0001	<0.0001	<0.0001	0.02
	Limbic gamma	2.85	0.08	2.84	0.13	2.50	0.07	4.34	0.34	772	>0.9999	0.56	<0.0001	.59	<0.0001	<0.0001
	Cognitive theta	5.29	0.06	4.83	0.06	5.38	0.07	7.33	0.05	772	<0.0001	0.72	<0.0001	<0.0001	<0.0001	<0.0001
	Motor beta	-0.38	0.07	-0.32	0.04	-0.40	0.04	1.00	0.14	3084	1.00	>0.9999	<0.0001	0.99	<0.0001	<0.0001

Supplementary Table 3: Cluster found on the SPM for M1 and M2. DL=dorsolateral, DL=dorsomedial, VL=ventrolateral, VM=ventromedial.
